# Nutritional Support Patterns and Outcomes in Pediatric Veno-Venous and Veno-Arterial Extracorporeal Membrane Oxygenation: A Retrospective Analysis

**DOI:** 10.3390/nu17243928

**Published:** 2025-12-16

**Authors:** Marwa Mansour, Nancy Chung, Blessy Philip, Kelly Martinek, Jesse Stoakes, Sarah Nelin, Nicole Knebusch, Cole Burgman, Jorge A. Coss-Bu, Andrea Ontaneda

**Affiliations:** 1 Division of Critical Care Medicine, Department of Pediatrics, Baylor College of Medicine, Houston, TX 77030, USA; marwa.mansour@bcm.edu (M.M.);; 2 Texas Children’s Hospital, Houston, TX 77030, USA; 3 Division of Critical Care, Department of Pediatrics, University of Texas Southwestern, Dallas, TX 75390, USA; nancy.chung@utsouthwestern.edu; 4 Division of Newborn Medicine, Department of Pediatrics, University of Mississippi Medical Center, Jackson, MS 39216, USA; 5 Imperial College of London, London SW7 2AZ, UK

**Keywords:** pediatric ECMO, critical care nutrition, circuit dysfunction, extracorporeal therapy, feeding intolerance, enteral nutrition, parenteral nutrition

## Abstract

Background: Nutritional support in patients receiving extracorporeal membrane oxygenation (ECMO) is a clinical challenge. Hemodynamic instability and concerns about gut perfusion delay enteral nutrition (EN), resulting in frequent use of total parenteral nutrition (TPN). This study aimed to compare nutritional practices in patients on venoarterial (VA) vs. venovenous (VV) ECMO, and to evaluate the associations between prolonged TPN use, feeding status, circuit change frequency, length of stay, and survival. Methods: Retrospective cohort study of ECMO patients in a quaternary pediatric intensive care unit. Nutritional variables included route and amount of nutrition delivery. The primary outcome was the nutrition type (enteral vs. parenteral) in association with ECMO mode (VV vs. VA). Secondary outcomes included associations between nutrition variables (TPN by Day 14, lack of EN by Day 5 or 7) and circuit changes, ECMO duration, ICU/hospital length of stay (LOS), and mortality. Analyses by Mann–Whitney and chi-square tests. Multivariable Poisson regression was used to identify independent predictors of circuit change frequency. Results: Patients on VV ECMO achieved higher enteral intake than those on VA ECMO. Persistent need for TPN by Day 14 was associated with longer PICU LOS, hospital LOS, and ECMO duration and was independently associated with 71% higher circuit change frequency. Survival did not differ significantly by TPN duration or early EN exposure. Conclusions: VV ECMO patients received higher enteral nutrition. Persistent need for TPN by day 14 was associated with worse outcomes. These findings underscore the need for standardized, evidence-based feeding strategies in this population.

## 1. Introduction

Optimal nutritional support is a fundamental aspect of care in critically ill patients, influencing outcomes such as infection risk, wound healing, length of stay, and mortality [1,2]. In this population, early and adequate enteral nutrition delivery has been associated with improved survival and functional recovery [3,4,5].

Enteral nutrition (EN) is generally preferred over parenteral nutrition (PN) as it preserves gut integrity, modulates the immune response, and reduces infectious complications. However, in patients requiring advanced organ support such as extracorporeal membrane oxygenation (ECMO), achieving adequate and timely nutrition remains a major clinical challenge [6,7].

ECMO provides temporary cardiopulmonary support for patients with severe cardiac or respiratory failure refractory to conventional management. Two main configurations are used: veno-venous (VV) ECMO, which supports gas exchange in patients with isolated respiratory failure, and veno-arterial (VA) ECMO, which provides both cardiac and respiratory support in cases of severe circulatory compromise [8,9]. While advances in technology and critical care practices have improved survival [10], optimizing nutritional support for patients on ECMO remains a significant clinical challenge with reported usage of PN in up to 80% of patients on ECMO [11,12]. The physiological alterations associated with ECMO such as systemic inflammation, altered hemodynamics, and changes in gastrointestinal perfusion can delay both the initiation and advancement of enteral feeding [13,14].

In particular, patients supported on VA ECMO are often perceived to be at higher risk for gut ischemia and feeding intolerance due to compromised bowel perfusion. As a result, clinicians may hesitate to initiate or advance EN, leading to heterogeneous nutritional practices and frequent suboptimal caloric and protein delivery throughout the ECMO course [6,11].

Although several studies have described feeding practices in ECMO populations, few have directly compared nutritional strategies between venovenous (VV) and veno-arterial (VA) ECMO configurations. Given the differences in underlying pathophysiology, respiratory failure in VV ECMO vs. combined cardiac and circulatory failure in VA ECMO, understanding how ECMO mode influences nutrition delivery is clinically relevant. The study aimed to address two clinically important questions; (1) Do children supported with VV vs. VA ECMO receive different patterns of enteral and parenteral nutrition? and (2) Are delayed enteral feeding and prolonged dependence on TPN associated with greater circuit dysfunction or worse clinical outcomes?

We aimed to identify barriers that may limit advancement of enteral feeding, thereby informing future feeding protocols and optimizing nutritional care for this high-risk population.

## 2. Materials and Methods

This is a retrospective cohort analysis of index admissions to the pediatric intensive care unit (PICU) at a single quaternary institution. We included pediatric patients (aged one month to 18 years) who required veno-venous (VV) or veno-arterial (VA) ECMO, from January 2015 through December 2024. Inclusion criteria were; 

(1) ECMO support for more than 7 days. (2) Patients received calculable nutrition intake by feeding tube or parenteral route. Patients with missing data, congenital heart disease, or post-cardiopulmonary bypass were excluded. The Institutional Review Board at Baylor College of Medicine, Houston (IRB: H-5692) approved this study’s ethical considerations in February 2025.

Data points were collected from information documented in the electronic medical record (EMR), encompassing patients’ demographics, admission anthropometric measurements [weight (kilograms), height/length (cm)] upon admission, PICU length of stay (LOS), hospital LOS, ECMO mode and duration, mortality, and nutrition-related variables such as route, volume, macronutrient intake, and documented barriers to initiating or advancing enteral feeds.

### 2.1. Nutrition Assessment

Anthropometric data were used to calculate z-scores for nutritional assessment. For patients younger than two years of age we utilized growth curves by the World Health Organization (WHO), and for those older than two years we utilized growth curves according to the Centers for Disease Control and Prevention (CDC) [15] Specifically, the anthropometric measures employed were weight-for-age (WFA), height-for-age (HFA) z scores, and body mass index-for-age (BMI/A) or weight-for-length (WFL) z scores.

Nutritional status was categorized as follows: underweight, WFA z score < −2.0; acute malnutrition, defined as a weight for height z score < −2.0; chronic malnutrition, HFA z score < −2.0; overweight, weight for height z score > 2.0; and obesity, weight for height z score > 3.0 [16,17].

All nutritional categories were based on age and sex.

### 2.2. Nutrition Support (Amount and Delivery Methods)

We collected data from the electronic medical record on nutritional variables, including macronutrient (calories and protein) prescriptions by the clinical dietitians and the medical team, actual daily caloric and protein delivery, route of delivery: enteral (EN) vs. parenteral (PN), and timing of the clinical dietitian’s evaluation. Nutritional intake was calculated on days 1, 3, 5, and 7, with day 0 being the day of ECMO initiation.

Using the Schofield equation, basal metabolic rate (BMR) was used to estimate the daily recommended caloric intake [18].

Goal protein intake was calculated as 2–3 g/kg for 0–2 years, 1.5–2 g/kg for 2–13 years, and 1.5 g/kg/day for 13–18 years [19]. We identified nutrition goal adequacy as 60% of the recommended daily caloric and protein intake and calculated it as [(intake/recommended) × 100] [20]. We compared nutritional adequacy and route of nutrition delivery across several subgroups, including ECMO mode (VV vs.VA ECMO), age (<2 years vs. >2 years), and nutritional status (underweight vs. non-underweight).

### 2.3. ECMO Outcomes

The primary outcome is the association between ECMO mode (VV vs. VA) and achieving goal nutrition by day 7 of ECMO support, via the enteral vs. parenteral route. 

Secondary outcomes are relationships between nutrition-related variables, including persistent need of TPN and receipt of TPN by Days 7 and 14, and absence of enteral nutrition during the first 5 or 7 days of ECMO support, and several clinically relevant endpoints. These endpoints included the total number of ECMO circuit changes, duration of ECMO support, ICU and hospital LOS, and survival to hospital discharge. A multivariable analysis included nutrition variables, ECMO type, age, and sex.

These nutrition variables were selected a priori based on their clinical relevance and established use in ECMO nutrition research. TPN use at Days 7 and 14 reflects sustained dependence on parenteral nutrition and serves as a practical marker of feeding intolerance and illness severity during the early ECMO course. Similarly, absence of enteral nutrition by Days 5 and 7 captures early feeding practices and aligns with guideline-recommended time windows for evaluating readiness for enteral feeding in critically ill children. Together, these variables represent key milestones in nutritional progression and provide meaningful insight into the interaction between feeding tolerance, ECMO physiology, and clinical outcome.

### 2.4. Statistical Analysis

Descriptive statistics were used to summarize patient demographics, nutritional variables, and clinical outcomes. Continuous variables were expressed as median (interquartile range) and compared using the Mann–Whitney U test. Categorical variables were summarized as counts and percentages and compared using the chi-square or Fisher’s exact test, as appropriate.

To examine the independent association between nutritional variables and ECMO circuit dysfunction (circuit change frequency), a multivariable Poisson regression model was constructed with circuit change frequency as the dependent variable. Independent variables included receipt of total parenteral nutrition (TPN) by Day 7, receipt of TPN by Day 14, and absence of enteral feeding during the first 5 days and first 7 days of ECMO support. Regression coefficients (β) were exponentiated to obtain incidence rate ratios (IRR) with 95% confidence intervals (CI). A *p*-value < 0.05 was considered statistically significant. All analyses were performed using Stat View Version 5.0.1 (SAS Institute Inc., Cary, NC, USA) and Python (v3.11, statsmodels package).

## 3. Results

During the study period, 115 patients met the inclusion criteria, of whom 51% were females. The median (interquartile range (IQR)) age was 3 years (1–11.75), and the median weight was 14.7 kg (8.9–38.8). The median ICU LOS was 46 days (26–75.8), and the median ECMO duration was 17 (9–30) days. Among the cohort, 20% received VA ECMO, and 80% received VV ECMO.

Primary indications for ECMO initiation included acute respiratory distress syndrome (ARDS) of mixed etiology (74%, n = 85), asthma (9.5%, n = 11), pulmonary hypertension (7.8%, n = 9), sepsis (4.3%, n = 5), air leak syndrome (1.7%, n = 2), and mediastinal mass (0.8%, n = 1).

A total of 53% (n = 61) of patients were evaluated by a dietitian within 48 h of ECMO initiation.

The prevalence of malnutrition categories was as follows: underweight (16%), obesity (15%), and chronic malnutrition (20%). Overall mortality for the cohort was 26% (Table 1).

### 3.1. Nutrition Support

**Figure 1:**
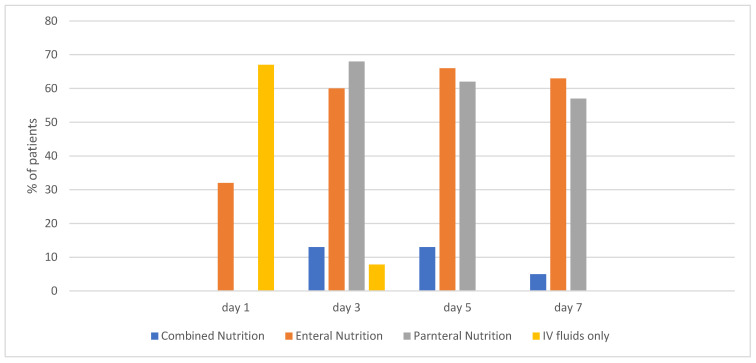
Percentage of patients receiving each type of Nutrition Support by day.

On day 1 of ECMO, 33% (n = 38) of patients were on an enteral diet. That percentage increased to 63% (n = 73) by day 7 (Figure 1).

By day 7 of ECMO, 30% of patients achieved goal nutrition adequacy (60% of protein and caloric needs) through EN. This percentage increased to ~80% of this cohort when calculating EN and PN intake.

**Figure 2:**
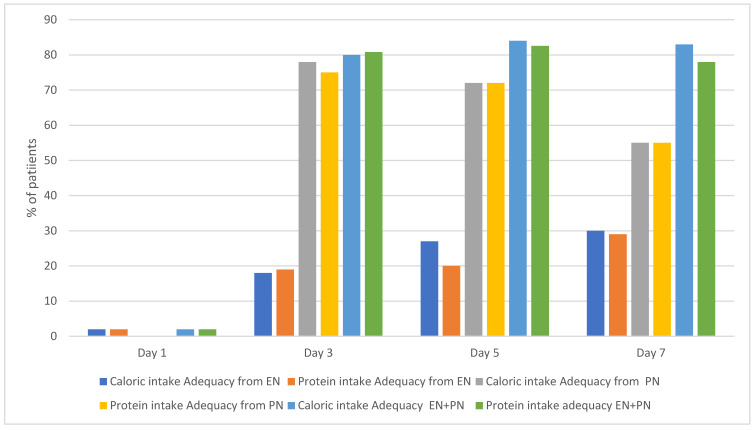
Percentage of patients who achieved caloric and protein intake adequacy.

The percentages of patients who achieved goal nutrition adequacy on EN alone, PN alone, and EN+PN on days 1, 3, 5, and 7 are shown in Figure 2.

Patients who had been receiving EN within 48 h prior to ECMO were more likely to remain on EN by day 1 of ECMO compared with those who were not previously enterally fed (47.5% vs. 16.7%; *p* < 0.0005). This difference was no longer significant by day 3 of ECMO (59% vs. 63%; *p* = 0.3).

#### 3.1.1. Nutritional Adequacy by ECMO Mode (VA vs. VV)

We compared the achievement of optimal nutrition adequacy between patients supported on VA ECMO (n = 27) and VV ECMO (n = 88).

On Day 3, patients on VA ECMO achieved significantly lower enteral protein and caloric intake than those on VV ECMO: 0 (0–9) vs. 10 (0–40), *p* = 0.02, and 0 (0–12) vs. 16 (10–50), *p* = 0.01, respectively. Similar trends were observed on day 5, 0 (0–26) vs. 11 (0–63) *p* = 0.07 and 11 (0–32) vs. 14 (0–76) *p* = 0.02 for protein and caloric intake adequacy, respectively (Table 2).

When total intake from combined EN and PN was analyzed, patients on VA ECMO received higher protein and caloric intake compared with those on VV ECMO (Table 2). On day 5, protein and caloric intake adequacy were 138 (115–160) vs. 108 (72–140) *p* = 0.04 and 126 (94–157) vs. 112 (81–140) *p* = 0.3, respectively. By day 7, protein adequacy remained higher in VA ECMO patients 135 (113–143) vs. 105 (57–143) *p* = 0.03, as did caloric adequacy 137 (104–168) vs. 111 (80–129) *p* = 0.002 (Table 2).

#### 3.1.2. Nutritional Adequacy by Age Groups (Age < vs. >2 Years Old)

In this cohort, no statistically significant differences were observed in EN provision practices between patients younger than 2 years and those 2 years or older.

However, patients under 2 years of age had higher caloric adequacy from EN+PN, 140 (108–167) vs. 103 (78–129), *p* = <0.001 on day 5, and 136 (115–168) vs. 106 (82–127), *p* < 0.001 on day 7. This difference was not observed for protein intake (Table 3).

#### 3.1.3. Nutritional Adequacy by Nutritional Status (Underweight vs. Non-Underweight Patients)

Comparing underweight and non-underweight patients, there was no difference in enteral protein and calorie intake adequacy between underweight and non-underweight patients on days 1, 3, and 5. Compared to underweight patients, non-underweight patients were more likely to receive higher caloric and protein intake enterally 0 (0–2) vs. 13 (0–62), *p* = 0.02 and 0 (0–7) vs. 24 (0–89), *p* = 0.04 for protein and caloric intake adequacy on day 7, respectively (Table 4).

The overall intake from EN+PN calculations showed higher caloric intake of underweight vs. non-underweight patients, 145 (123–177) vs. 111 (18–136) *p* = 0.006 and 140 (88–177) vs. 115 (68–135) *p* = 0.09 on day 5 and 7, respectively. There was a statistically significant difference in protein intake between these two groups (Table 4).

### 3.2. Outcomes in Association with Nutrition Practices

In a multivariable Poisson regression model including all nutritional variables, ECMO type, sex and age, we found that the persistent need of TPN by Day 14 was independently associated with an increased rate of circuit changes (IRR 1.71, 95% CI 1.19–2.47, *p* = 0.004).

Other nutritional variables, including TPN by Day 7 and absence of enteral feeding during the first 5 or 7 days, were not significantly associated with circuit change frequency (Table 5).

Patients receiving TPN on day 14 of ECMO (44%, n = 51) had significantly longer PICU LOS compared to those who did not receive TPN, 58.0 (39.0–77.5) vs. 38.5 (20.8–66.5) days, *p* = 0.007, longer hospital LOS 77.6 (45.7–103.6) vs. 50.3 (29.0–83.0) days, *p* = 0.006, and longer ECMO duration 21.3 (13–32) vs. 13 (8–22.6) days, *p* = 0.007 (Table 6).

No statistically significant difference in mortality was observed between patients who received EN at any time during the first 5 days of ECMO and those who did not. The same finding was observed when comparing patients who received EN at any time during the first 7 days vs.those who did not (Table 6).

## 4. Discussion

In this cohort of pediatric patients on ECMO, ~30% achieved the nutritional adequacy goal, using EN alone, by the end of the first week of ECMO. At the same time, ~80% of patients achieved the same goal with EN+PN.

These findings are not consistent across VA and VV ECMO patients. Our analysis demonstrated a clear disparity in nutritional practices between the two modes: patients supported with VV ECMO were significantly more likely to achieve nutritional adequacy via the enteral route than those on VA ECMO. This difference underscores the persistent challenge of safely initiating and advancing enteral nutrition in patients receiving VA ECMO, who often have greater hemodynamic instability and higher risk of feeding intolerance [6,11].

In contrast, patients on VA ECMO in this study achieved greater overall nutritional adequacy when considering combined EN and PN delivery. On average, VA ECMO patients received 135% of their protein goals and 137% of their caloric goals, compared with 105% and 111%, respectively, among VV ECMO patients. This observation raises important questions regarding how nutritional requirements are assessed in VV vs. VA ECMO patients, how underfeeding and overfeeding should be defined, how best to quantify caloric and protein delivery in this complex population, and how is the amount of nutrition delivery associated with clinical outcomes e.g., mortality.

In a study of adult patients on VV ECMO, higher protein intake during the first 14 days of therapy was independently associated with reduced mortality, highlighting the potential importance of early protein optimization in this population. In contrast, total energy intake over the same period was not significantly associated with clinical outcomes, suggesting that protein provision may play a more critical role than caloric delivery during the early course of ECMO support [21].

Despite the growing evidence that EN is associated with lower mortality and less PICU LOS, initiating and advancing enteral diet, especially in patients on VA ECMO, remains a challenge as shown in our analysis [5,22,23,24,25].

Advancing EN in critically ill patients is often hindered by a combination of physiological, clinical, and institutional barriers. Hemodynamic instability and high vasoactive requirements raise concerns about mesenteric hypoperfusion and the risk of intestinal ischemia [26].

As a result, many ECMO patients, especially those on VA support, experience delayed advancement to goal enteral nutrition despite emerging evidence supporting its safety and benefits in hemodynamically stable patients [6,11,27].

In this cohort of patients, gastric intolerance, emesis, and abdominal distention are commonly documented reasons for stopping the feeds and often lead to cautious advancement or prolonged reliance on PN with or without enteral trophic feeding. Additionally, we found frequent interruptions for diagnostic and therapeutic procedures and imaging studies that halt or at least delay the progression of feeds.

Interestingly, in our analysis, we found that patients who were on an enteral diet within 2 days before ECMO initiation were more likely to receive an enteral diet on Day 1 of ECMO, which might be explained by a higher provider comfort level to initiate enteral diet, given the recent history of enteral feeding intolerance.

Gut failure is common in critically ill patients and reflects the complex interplay of shock, inflammation, and impaired splanchnic perfusion [28,29]. Because ECMO is reserved for patients who have already failed conventional therapies [8,9], these patients are initiated on ECMO support with significant physiologic instability. Moreover, ECMO itself can trigger a systemic inflammatory response and alter gut perfusion, further compromising intestinal barrier integrity [13,14,29]. As a result, patients supported with ECMO are at particularly high risk for gut dysfunction and its associated complications. This hypothesis has been studied in preclinical studies. In a neonatal porcine model, investigators reported that initiating ECMO in healthy piglets led to intestinal barrier dysfunction and bacterial translocation, resulting from increased gut permeability. The authors proposed that barrier dysfunction may serve as a primary initiator or amplifier of ECMO-related systemic inflammatory response syndrome (SIRS), rather than merely representing a secondary epiphenomenon associated with loss of mucosal integrity as part of ECMO-induced multisystem dysfunction [30]. In a clinical study of neonates, Piena et al. demonstrated that intestinal integrity is impaired in infants supported with ECMO and that the introduction of enteral nutrition did not further deteriorate intestinal integrity. This notable finding challenges the long-standing concern that feeding may exacerbate gut injury. Instead, it suggests that enteral nutrition can be safely initiated in critically ill neonates receiving ECMO support [31].

Despite the lack of clinical trials, many observational studies have shown safe enteral diet in ECMO patients [4,32,33]. Hence, enteral nutrition, when appropriate, is recommended by guidelines [3,19,27,34].

In our study, reasons for delaying or pausing feeds included constipation, abdominal distension, emesis, and nausea. No severe gastrointestinal complications, e.g., necrotizing enterocolitis, bowel ischemia, etc., were documented in relation to the enteral diet.

These findings call for larger, prospective, randomized studies to examine gut barrier integrity markers, enteral diet feasibility, safety, and outcomes in this heterogeneous population.

When EN is not achievable, parenteral nutrition is often used to augment nutritional intake [12,35]. The literature shows better clinical outcomes in PICU patients when 60% of caloric and protein needs are met in the first week [2,19,36]. Clinicians may find it difficult to achieve this goal with EN alone. Hence, PN is widely used [12,35].

In our analysis, 80% of the cohort achieved the nutritional goals when TPN intake was combined with EN. The primary concern with overutilizing TPN is its side effect profile, i.e., immune dysregulation, microbiome dysfunction, increased risk of sepsis, electrolyte imbalance, and liver dysfunction [37,38,39,40,41].

The effects of delayed enteral nutrition and prolonged parenteral nutrition use during ECMO remain largely unexamined. An observational study in adults on ECMO reported a higher risk of circuit dysfunction among patients on PN [40].

In our study, patients with persistent PN requirements during the first 2 weeks of ECMO had a higher rate of circuit changes (IRR 1.76, 95% CI 1.22–2.52, *p* = 0.002).

Although statistically significant, this finding does not establish causation or independent association, as other factors, such as severe sepsis, multiorgan failure, immune dysregulation, and coagulopathy, may confound the results. Prospective large-scale studies are needed to explore the relationships among TPN, inflammation, immune dysregulation, coagulopathy, and circuit dysfunction in ECMO patients.

The literature shows better outcomes in patients who tolerate early enteral nutrition compared to those who don’t [5,25,32]. In this study, patients with persistent need for PN, had longer Hospital LOS, PICU LOS, and ECMO LOS. There was no difference in mortality in relation to different nutrition provision status (Table 6).

These findings have several practical implications for clinical nutrition management in ECMO patients. Identifying patients at risk for delayed enteral feeding—particularly those supported with VA ECMO—may allow earlier implementation of targeted nutrition strategies. For example, early stratification based on ECMO mode, hemodynamic stability, and feeding tolerance in the first 48–72 h could help determine which patients may benefit from more proactive TPN initiation vs. cautious but earlier EN trials. Our results also suggest that persistent TPN dependence by Day 14 is associated with greater circuit instability, highlighting the importance of close monitoring and timely reassessment of feeding readiness. Integrating these insights into structured feeding pathways may support more consistent decision-making around EN advancement, reduce unnecessary delays in enteral initiation, and help mitigate the metabolic consequences of prolonged underfeeding. Ultimately, applying individualized nutrition strategies informed by ECMO configuration and early feeding patterns may improve both nutritional adequacy and broader clinical outcomes.

## 5. Conclusions

In this study, we observed that patients supported with VV ECMO were more likely to receive and advance enteral nutrition than those on VA ECMO. The need for prolonged parenteral nutrition, particularly by Day 14, was associated with a higher rate of circuit dysfunction, longer ECMO duration and extended ICU and hospital stays, although not with increased mortality.

Early enteral feeding initiation remained limited, and a significant proportion of patients did not achieve enteral nutrition within the first week of ECMO. These findings underscore the challenges of achieving adequate nutrition during ECMO support and highlight the need for objective criteria to guide safe advancement of enteral nutrition in this population.

Future research should aim to identify strategies to safely advance enteral nutrition, reduce dependence on parenteral support, and better understand the metabolic and inflammatory consequences of varying nutrition practices in ECMO patients.

### Limitations

This study has several limitations. First, its retrospective single-center design limits the ability to infer causality and may introduce selection and documentation bias. Second, variability in feeding practices among providers and the absence of a standardized feeding protocol could have influenced the timing and advancement of nutrition. Although our multivariable model adjusted for several nutrition-related and demographic factors, we were unable to control for important clinical confounders such as illness severity scores, vasoactive support, and underlying diagnosis because these variables were not consistently available in our retrospective dataset. Finally, because this analysis was limited to patients with complete nutritional and ECMO data, the findings may not be generalizable to all ECMO populations or to other institutions.

## Figures and Tables

**Table 1:** Patient Demographics for Nutrition Support.

n	115
Female, % (n)	51% (59)
Age (years)	3 (1–11.75)
Weight (kg)	14.7 (8.9–38.8)
Height (cm)	98.2 (74.3–145.5)
PICU LOS Physical (days)	46 (26–75.8)
LOS ECMO (days)	16.6 (9–30.5)
VA ECMO, % (n)	20 (23)
VV ECMO, % (n)	80 (92)
Circuit change occurrences	2 (0–7)
Underweight, % (n)	16 (14)
Chronic malnutrition, % (n)	20 (23)
Acute Malnutrition, % (n)	11 (13)
Obesity, % (n)	15 (17)
Mortality, % (n)	26 (30)

Continuous variables are represented as medians with interquartile ranges (25–75th). Categorical variables are expressed as numbers and percentages. ECMO: Extra Corporeal Membrane Oxygenation; PICU LOS: Pediatric Intensive Care Unit Length of Stay.

**Table 2:** Protein and caloric adequacy in VA vs. VV patients.

**Enteral Nutrition**						
	**Protein Adequacy**			**Caloric Adequacy**		
	**VA** **n = 27**	**VV** **n = 88**	***p* Value**	**VA** **n = 27**	**VV** **n = 88**	***p* Value**
Day 1	0	0	0.1	0	0	0.13
Day 3	0 (0–9)	10 (0–40)	0.02	0 (0–12)	16 (10–50)	0.01
Day 5	0 (0–26)	11 (0–63)	0.07	11 (0–32)	14 (0–76)	0.02
Day 7	2 (0–62)	9 (0–57)	0.8	25 (0–105)	80 (16–126)	0.9
**Enteral Nutrition + Parenteral Nutrition**						
	**Protein Adequacy**			**Caloric Adequacy**		
	**VA** **n = 27**	**VV** **n = 88**	***p* Value**	**VA** **n = 27**	**VV** **n = 88**	***p* Value**
Day 1	0	0 (0–4)	0.1	0 (0–12)	0 (0–16)	0.01
Day 3	162 (90–143)	120 (62–152)	0.5	111 (88–166)	118 (80–143)	0.9
Day 5	138 (115–160)	108 (72–140)	0.04	126 (94–157)	112 (81–140)	0.3
Day 7	135 (113–143)	105 (57–143)	0.03	137 (104–168)	111 (80–129)	0.002

Continuous variables are represented as medians with interquartile ranges (25–75th). Adequacy % = (amount taken/amount prescribed) × 100. VV: Veno-venous. VA: Veno-arterial. Mann-Whitney test was utilized to compare two independent groups with continuous variables.

**Table 3:** Protein and caloric adequacy in <2 years vs. >2 years patients.

**Enteral Nutrition**						
	**Protein Adequacy**			**Caloric Adequacy**		
	**<2 Years** **n = 40**	**>2 Years** **n = 75**	***p* Value**	**<2 Years** **n = 40**	**>2 Years** **n = 75**	***p* Value**
Day 1	0 (0–2)	0 (0–0.3)	0.5	0 (0–6)	0 (0–0.2)	0.4
Day 3	4 (0–50)	5 (0–26)	0.4	16 (0–90)	7 (0–43)	0.23
Day 5	7 (0–29)	5 (0–65)	0.9	12.5 (0–52)	8 (0–72)	0.5
Day 7	9 (0–55)	5 (0–60)	0.4	13 (0–117)	12 (0–73)	0.1
**Enteral Nutrition + Parenteral Nutrition**						
	**Protein Adequacy**			**Caloric Adequacy**		
	**<2 Years** **n = 40**	**>2 Years** **n = 75**	***p* Value**	**<2 Years** **n = 40**	**>2 Years** **n = 75**	***p* Value**
Day 1	0 (0–3)	0 (0–0.3)	0.5	16 (0–90)	6 (0–43)	0.23
Day 3	120 (99–133)	132 (60–100)	0.8	139 (119–172)	104 (59–128)	0.001
Day 5	116 (93–133)	125 (72–158)	0.3	140 (108–167)	103 (78–129)	<0.001
Day 7	112 (60–137)	126 (67–148)	0.19	136 (115–168)	106 (82–127)	<0.001

Continuous variables are represented as medians with interquartile ranges (25–75th). Adequacy % = (amount taken/amount prescribed) × 100. Mann-Whitney test was utilized to compare two independent groups with continuous variables.

**Table 4:** Protein and caloric adequacy in underweight vs.non-underweight patients.

**Enteral Nutrition**						
	**Protein Adequacy**			**Caloric Adequacy**		
	**Underweight n = 16**	**Non-Underweight n = 99**	***p* Value**	**Underweight n = 16**	**Non-Underweight n = 99**	***p* Value**
Day 1	0 (0–9)	0 (0–0.7)	0.21	0 (0–22)	0 (0–0.2)	0.15
Day 3	8 (0–50)	6 (0–23)	0.8	5 (0–79)	9 (0–45)	0.9
Day 5	2 (0–20)	10 (0–65)	0.2	1 (0–24)	13 (0–73)	0.21
Day 7	0 (0–2)	13 (0–62)	0.02	0 (0–7)	24 (0–89)	0.04
**Enteral Nutrition + Parenteral Nutrition**						
	**Protein Adequacy**			**Caloric Adequacy**		
	**Underweight n = 16**	**Non-Underweight n = 99**	***p* Value**	**Underweight n = 16**	**Non-Underweight n = 99**	***p* Value**
Day1	0 (0–9)	0 (0–0.7)	0.2	5 (0–79)	9 (9–45)	0.9
Day 3	124 (41–157)	121 (80–150)	0.9	114 (87–141)	136 (59–182)	0.2
Day 5	129 (104–149)	116 (73–147)	0.4	145 (123–177)	111 (18–136)	0.006
Day 7	130 (71–138)	113 (66–144)	0.9	140 (88–177)	115 (68–135)	0.09

Continuous variables are represented as medians with interquartile ranges (25–75th). Adequacy % = (amount taken/amount prescribed) × 100. Mann-Whitney test was utilized to compare two independent groups with continuous variables.

**Table 5:** Multivariable Poisson Regression Assessing the Association Between Nutritional Variables and Circuit Change Frequency in ECMO Patients.

	Coefficient (β)	IRR	95% CI for IRR	*p*-Value
TPN by Day 7	−0.213	0.81	0.56–1.16	0.25
TPN by Day 14	0.563	1.71	1.19–2.47	0.004
Never on EN during first 5 days	−0.858	0.42	0.17–1.04	0.06
Never on EN during first week	0.383	1.47	0.55–3.93	0.45

IRR: incidence rate ratios. Circuit Change Frequency is a continuous variable. Other variables are binary variables.

**Table 6:** Clinical outcomes in association with TPN intake on Day 14 of ECMO:.

	TPN Day 14; Yes n = 51	TPN Day 14; No n = 64	*p* Value
ICU LOS, days	58.0 (39.0–77.5)	38.5 (20.8–66.5)	0.007
Hospital LOS, days	77.6 (45.7–103.6)	50.3 (29.0–83.0)	0.006
ECMO LOS, days	21.3(13–32)	13 (8–22.6)	0.007
Mortality, % (n)			
	TPN Day 14; Yes n = 51	TPN Day 14; No n = 64	
	23.5 (12)	28 (18)	0.58
	Never on EN during first 5 days; Yes n = 27	Never on EN during first 5 days; No n = 88	
	30 (9)	24 (21)	0.47
	Never on EN during first 7 days; Yes n = 19	Never on EN during first 7 days; No n = 69	
	42 (8)	23 (22)	0.15

Continuous variables are represented as medians with interquartile ranges (25–75th). Categorical variables are expressed as numbers and percentages. Analysis by Chi-Square test for comparisons of categorical variables. Mann-Whitney test was utilized to compare two independent groups with continuous variables.

## Data Availability

The raw data supporting the conclusions of this article will be made available by the authors on request.

## Abbreviations

The following abbreviations are used in this manuscript:

ECMO	extracorporeal membrane oxygenation
VA	venoarterial
VV	venovenous
EN	enteral nutrition
PN	parenteral nutrition
TPN	total parenteral nutrition
